# Effects of Culture Substrate Made of Poly(*N-*isopropylacrylamide-co-acrylic acid) Microgels on Osteogenic Differentiation of Mesenchymal Stem Cells

**DOI:** 10.3390/molecules21091192

**Published:** 2016-09-09

**Authors:** Zhuojun Dai, Yinglan Shu, Chao Wan, Chi Wu

**Affiliations:** 1 Department of Chemistry, The Chinese University of Hong Kong, Shatin, N.T., Hong Kong, China; chiwu@cuhk.edu.hk; 2 Ministry of Education Key Laboratory for Regenerative Medicine, School of Biomedical Sciences, Faculty of Medicine, The Chinese University of Hong Kong, Shatin, N.T., Hong Kong, China; shuyinglan88@gmail.com

**Keywords:** microgels, p(*N*-isopropylacrylamide-co-acrylic acid), mesenchymal stem cells, extracellular matrix, cell morphology, osteogenesis

## Abstract

Poly(*N*-isopropylacrylamide) (PNIPAM)-based polymers and gels are widely known and studied for their thermoresponsive property. In the biomaterials category, they are regarded as a potential cell culture substrate, not only because of their biocompatibility, but also their special character of allowing controlled detachment of cells via temperature stimulus. Previous research about PNIPAM-based substrates mostly concentrated on their effects in cell adhesion and proliferation. In this study, however, we investigate the influence of the PNIPAM-based substrate on the differentiation capacity of stem cells. Especially, we choose P(NIPAM-AA) microgels as a culture dish coating and mesenchymal stem cells (MSCs) are cultured on top of the microgels. Interestingly, we find that the morphology of MSCs changes remarkably on a microgel-coated surface, from the original spindle form to a more stretched and elongated cell shape. Accompanied by the alternation in morphology, the expression of several osteogenesis-related genes is elevated even without inducing factors. In the presence of full osteogenic medium, MSCs on a microgel substrate show an enhancement in the expression level of osteopontin and alizarin red staining signals, indicating the physical property of substrate has a direct effect on MSCs differentiation.

## 1. Introduction

Tissue engineering has tried to create functional replacements for tissues damaged by injury or disease via a combination of cells, scaffolds, and soluble mediators [1,2,3]. Particularly, unique properties of self-renewal and potential differentiation into multiple cell types lead stem cells to be a promising cell source for regenerative medicine therapies [4,5]. Among them, mesenchymal stem cells (MSCs) have been mostly used due to their remarkable characteristics of self-replication and multilineage differentiation capacity [6,7,8].

In a tissue engineering process, a number of factors influence the stem cell differentiation, including the in vivo and in vitro regulations by genetic and molecular mediators (e.g., the growth and transcription factors) [9]. However, some recent evidence has demonstrated that physical interactions between stem cells and the extracellular matrix (ECM) also affect the cell fate [9,10,11,12,13]. Discher and coworkers showed that MSCs responded to different elasticities of the ECM by differentiating into multiple cell types [14,15]. Besides the elasticity, material surface properties, such as the chemical composition and topography, also play some roles in both two- and three-dimensional cell culture [16,17].

On the other hand, it has been well known that cell attachment is the first major and essential step for later cell behaviors. Therefore, various cell culture substrates have been investigated and developed to facilitate cell attachment and digestion. Especially, some thermally sensitive polymers and their derivatives with a switchable/reversible hydrophilic-hydrophobic transition are widely explored as cell culture substrates [18,19]. Among them, poly(*N*-isopropylacrylamide) (PNIPAM), with a lower critical solution temperature (LCST ~ 32 °C), is the mostly studied system [20,21]; namely, at temperatures higher than ~32 °C, PNIPAM becomes insoluble in water. Therefore, the substrate will facilitate or resist cell adhesion when the temperature is above or below its LCST. Such a physical property has been exploited to recover those adherent cells from their substrates in a non-invasive way [22,23,24]. Intact cell sheets from these thermally sensitive surfaces were successfully obtained simply by reducing the temperature instead of by applying the enzymatic treatment [25,26]. Recently, Higuchi and coworkers successfully demonstrated the continuous harvest of stem cells, including human adipose-derived stem cells (hADSCs) and embryonic stem cells (hESCs), via partial detachment by applying thermoresponsive nanobrush surfaces [19]. The work shows advantages in labor and cost-saving compared with the traditional lab batch-type culture. Note that after altering the original cell culture substrate to a thermally sensitive one, one changes both chemical and physical properties of the surface even though most of the polymers used as biomaterials are chemically inert and have less direct effect on cell behavior. However, a switch of the surface from hydrophilic to hydrophobic influences the cells’ behavior since the cells are able to sense and respond to the physical signals [13,27]. A poly(*N*-isopropylacrylamide-*co*-acrylic acid) (P(NIPAM-AA))-coated surface is a typical example which has a switchable hydrophilicity, variable stiffness and designed topology, very different from a normal culture dish surface [28,29]. In the current study, we purposely coated culture dishes with P(NIPAM-AA) microgels with series of AA contents and cultured MSCs on different coated surfaces. With less AA content, the microgel is more hydrophobic, which is proved by the decrease of the LCST and more neutral zeta-potential at the condition of pH 7 [30]. Each microgel is a very tiny three-dimensional network made of inter-connected polymer chains. P(NIPAM-AA) microgels are dually pH and thermally sensitive, which enables us to study how the micro-environment affects the stem cells’ morphology and differentiation capacity.

## 2. Results and Discussion 

### 2.1. Coating of Microgels on Surface

**Figure 1:**
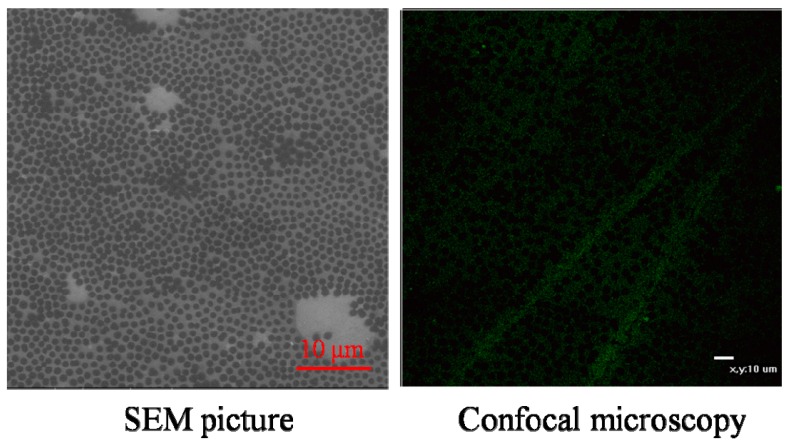
Typical imaging of a silica wafer (**left**) coated and a coverslip (**right**) with spherical poly(*N*-isopropylacrylamide-co-acrylic acid) (P(NIPAM-AA)) microgels, where the scale bar is 10 μm. The AA content is 10%.

The copolymerization of NIPAM and AA leads to spherical microgels with both thermal and pH sensitivities. As shown before [30], the microgels gradually swell as the temperature decreases. At pH 7, the ζ potential decreases as the AA content increases. The hydrophilicy of the coating will increase with the AA content. Note that the microgels used in the current study have an average size in the range of 1.5–2 μm at ~37 °C. Figure 1 shows the surface morphology of a silica wafer and a coverslip coated with the microgels. It shows that the microgels are spherical with a uniform size and densely packed to form a new substrate.

### 2.2. Effect of Microgel Coating on Morphology of MSCs

**Figure 2:**
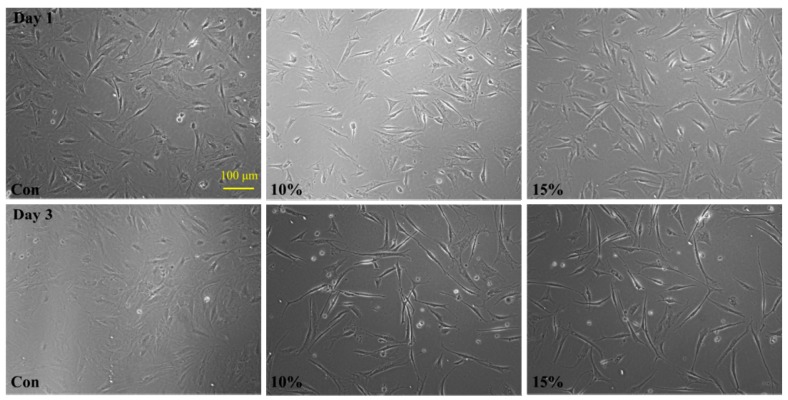
Morphological change of MSCs seeded on a culture dish coated with different microgels: The first and second rows show morphologies of MSCs on control (normal dish, **left**), P(NIPAM-AA) microgels with 10% (**center**) and 15% (**right**) AA contents after one and three days, respectively.

MSCs were first placed on a culture dish coated with the microgels (Day 0) and then supplemented with control medium (α-MEM). Figure 2 shows that MSCs stretch and elongate in comparison with their original spindle shape on a normal culture dish after 24 and 72 h, respectively. The change in the morphology is more obvious in the 10% and 15% AA content microgel substrate, probably due to the higher hydrophobicity of the surface.

**Figure 3:**
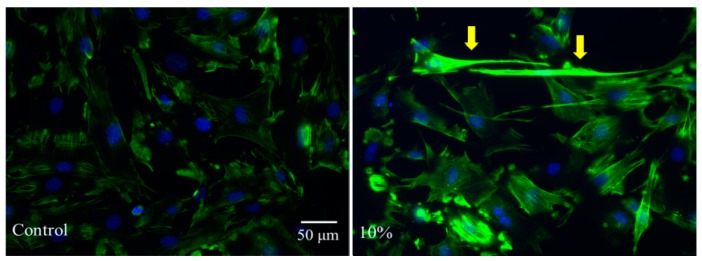
Immunostaining of F-actin in MSCs cultured on a normal dish (**left**) or a 10% microgel-coated dish (**right**), where arrows point to MSCs with enhanced fluorescence, i.e., an increased local density of actin filaments.

To check whether such a morphological change leads to the rearrangement of the cytoskeleton, we performed immunostaining for F-actin, a marker for actin filament, as displayed in the green color in Figure 3. It is clear that the cytoskeleton of MSCs is re-aligned on the microgel-coated substrate. Especially the local density of the F-actin filaments (marked by the arrow) in the stretched cells is dramatically increased, indicating the enhanced tension inside MSCs.

### 2.3. Effect of Morphology Change on MSCs’ Fate without Any Inducing Factors

**Figure 4:**
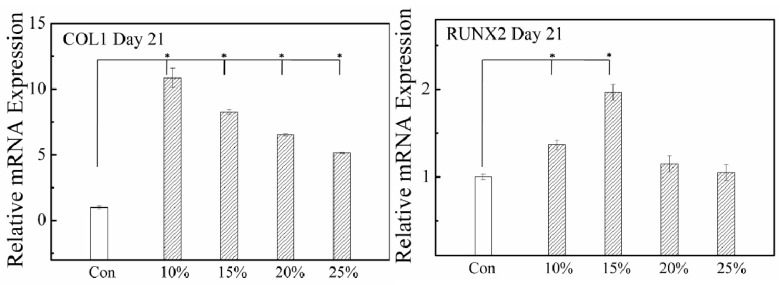
Osteogensis-related gene expression of MSCs on substrates coated with different microgels after 21 days with no inducing factor supplemented. Con denoted as a common culture dish; 10%, 15%, 20%, and 25% indicate coating of P(NIPAM-AA) microgels with different AA contents, respectively. For all the substrates, the medium contains no inducing factor. * *p* < 0.05 and *n* = 3.

To elucidate a possible correlation between the morphological change and the cell fate, we cultured MSCs on different substrates using a normal cell culture medium without any inducing factors up to 21 days. For MSCs cultured on the microgel-coated substrate, the Alizarin Red S staining showed a totally negative signal (data not shown), suggesting that ECM mineralization did not occur because of the lack of osteogenic supplements. However, Figure 4 shows significantly increased mRNA levels of two osteogenic marker genes, RUNX2 and COL1, indicating that the morphological change has a positive effect on osteogenesis. Especially the increment is more remarkable for cells growing on a more hydrophobic surface, which is in accordance with the morphological change.

### 2.4. Effect of Morphological Change on MSCs’ Fate with Inducing Factors

**Figure 5:**
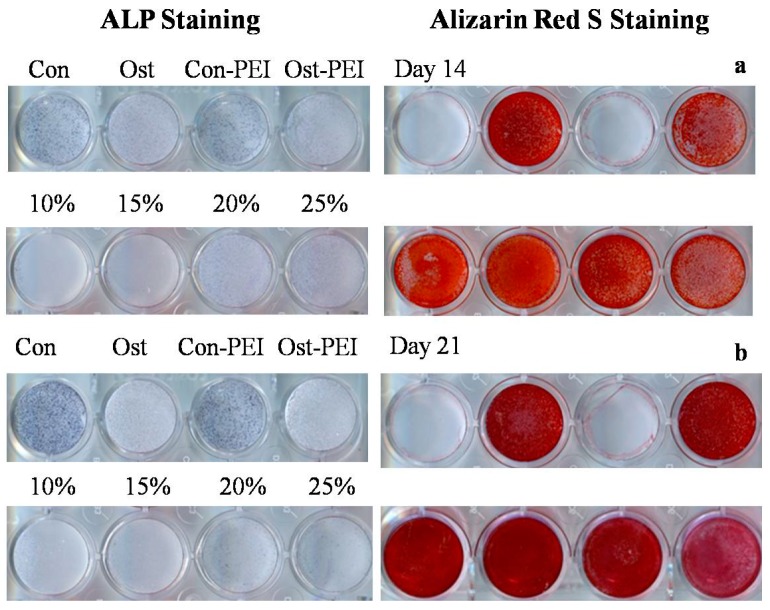
Effects of different substrates on osteogenic differentiation of MSCs in a full osteogenic medium after 14 and 21 days, respectively, using ALP and Alizarin Red S staining. In both (**a**,**b**), the first and second rows display different medium treatments, respectively, on substrates without and with microgel coatings, where Con denotes a normal medium on a normal culture dish; Ost, a full osteogenic medium on a normal culture dish; Con-PEI, a normal medium on a normal culture dish coated only with PEI; and Ost-PEI, a full osteogenic medium on a normal culture dish coated with PEI. The 10%, 15%, 20%, and 25% indicate coating of P(NIPAM-AA) microgels with different AA contents, respectively, and are treated by full osteogenic medium.

After confirming the enhancement of the osteogensis-related gene expression on the substrate coated with the microgels even without any inducing factors, we realize that the coating made of the microgels alone is not capable of leading to the mineralization and full osteogenic differentiation. Therefore, we have to incorporate some osteogenic inducing factors into the culture medium and comparatively study the effect of the microgel coating on the full osteogenic differentiation of MSCs. Figure 5 shows that after MSCs were planted on different substrates and cultured for different times in the full osteogenic medium, (1) the mineralization degree increased with the culture time; and (2) MSCs cultured on the microgel-coated substrate have a comparable or better mineralization degree in comparison with those on a common culture dish surface.

**Figure 6:**
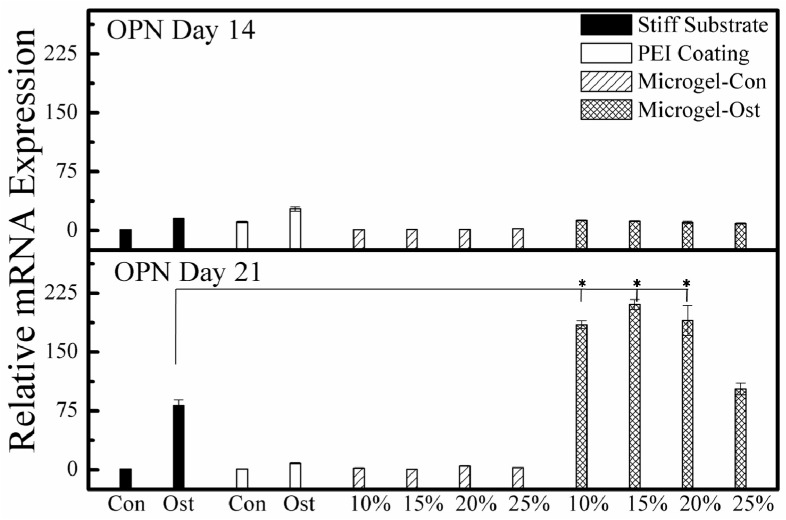
Medium- and substrate-dependent OPN expression of MSCs after different culture times, where Con and Ost are respectively denoted to a normal or a full osteogenic medium treatment on MSCs planted on a normal (filled columns) or an only PEI-coated (blank column) culture dish; and two types of shadowed columns represent a normal or an osteogenic medium treatment of MSCs planted on substrates coated with microgels. The 10%, 15%, 20%, and 25% indicate coating of P(NIPAM-AA) microgels with different AA contents, respectively, and are treated by full osteogenic medium. * *p* < 0.05 and *n* = 3.

We further checked the gene expression of alkaline phosphatase (ALP) and osteopontin(OPN). It has been known that the in vitro osteogenesis closely resembles the in vivo bone formation, experiencing three different stages [7,31]. In the first stage (Days 1–4), there is a remarkable increase in the cell number. The second early osteoblastic differentiation stage (Days 5–14) is characterized by the transcription and protein expression of ALP. In the third late stage (Days 14–28), the expression levels of ALP gradually decrease and the mature osteoblast marker OPN is highly expressed together with the depositions of calcium and phosphate. Figure 6 clearly shows that the expression of OPN increases in the presence of the inducing factors in comparison with that under the normal culture environment. It is worth noting that after 21 days, the expression of OPN on the microgel-coated dish, especially those coated by more hydrophobic microgels, prevails over the full osteogenic medium treatment on a normal dish, which is also in accordance with the Alizarin Red S staining result, indicating that the morphological change of MSCs, especially an increase in the tension in the intracellular space, is potentially helpful in promoting osteogensis.

It has been known that some properties of a material affect the cell adhesion, including its texture, water content, rigidity and hydrophobicity [28]. In fact, these properties also influence the cell proliferation and morphology changes. In the current study, the hydrophobicity, rigidity, texture and pattern of a substrate coated with spherical P(NIPAM-AA) microgels are different from a normal culture dish. The soft and hydrophobic surface limits the migration of MSCs, resulting in a more stretched and elongated cell shape. On the other hand, the cell morphology is an essential marker in phenotype and is related to the cell’s specialized functions. Starting from the same precursor, adipocytes are round and fat-laden but osteoblasts vary from elongated to cuboidal since the spherical shape allows maximal lipid storage in adipose tissue, while the spreading facilitates the osteoblast matrix deposition during bone formation. Using some micro-patterned islands on an ECM to control the cell spreading, McBeath et al. showed that the cell shape actually controled the lineage commitment of MSCs [32].

Different cell morphologies must arise from different expression of integrins, cadherins, and cytoskeletal proteins during the stem cell fate commitment. Especially the cytoskeleton plays a relatively important role and is primarily composed of three components: actin filaments, intermediate filaments and microtubules. F-actin molecules, the backbone of the cytoskeleton, cluster themselves to form an actin filament network and determine the mechanical properties of the living cells [33,34]. Stem cells integrate their micro-environmental stimulus, including soluble factors, adhesive contexts and mechanical signals to response, in a relevant differentiation.

In spite of the cell shape being closely correlated to biological processes including cell proliferation and differentiation, the underling molecular mechanisms of the phenotypes observed remain to be elucidated. It was mentioned in previous studies that the parallel cytoskeleton with thin actin fibers becomes disordered and robust with thicker stress fibers when MSCs undergo osteogenic differentiation [35], consistent with our current results. Therefore, we propose that the substrates coated with soft and spherical microgels provide a proper mechanical property that alters the F-actin alignment and the cell shape, and subsequently affects the differentiation of MSCs.

## 3. Materials and Methods

### 3.1. Materials

*N*-isopropylacrylamide (NIPAM, Fluka, St. Louis, MO, USA) was recrystallized from toluene/*n*-hexane. Acrylic acid (AA, Adrich), *N*,*N’*-methylenebis acrylamide (MBA, Fluka) and potassium persulfate (KPS. Merck, Darmstadt, Germany) were used as received without further purification. Deionized water was used in all the experiments.

### 3.2. Synthesis and Characterization of P(NIPAM-AA) Microgel Particles

Both the synthesis and characterization has been reported before [30]. For the benefit of readers, we briefly outline it as follows. AA and NIPAM monomers with different ratios (10%–25%) and cross-linking agent, *N*,*N’*-methylenebisacrylamide (MBA), were dissolved in deionized water. The mixture was stirred and purged with nitrogen for 30 min to remove oxygen. The reaction was initiated by potassium persulfate (KPS) and carried on for 6 h at 70 °C under the flow of nitrogen. The product was purified by centrifugation at 8500 *g* and re-suspended. The temperature sensitivity of the resultant microgels was characterize by using a laser light scattering (LLS) spectrometer (ALV/DLS/SLS-5022F) equipped with a multi-τ digital time correlator (ALV5000) and a cylindrical 22 mW He-Ne laser (λ_0_ = 632 nm, UNIPHASE). The pH sensitivity of the resultant microgels was characterized by the ζ potential measurement at 25 °C by using the ZetaPlus (Brookhaven Instruments Co., Holtsville, New York, NY, USA). In the low concentration range (<20 mg/mL), the microgels are nearly non-cytotoxic to MSCs and harmless in MSCs proliferation [30].

### 3.3. Coating Culture Dish with Microgels

Each culture dish was first coated with a solution of branched polyethylenimine (PEI, 25,000 g/mol, Sigma-Aldrich) (15 mg/L, in autoclaved water) for 30 min at room temperature; then the PEI solution was removed and the culture dish coated with PEI was washed with autoclaved water three times; and finally a microgels dispersion (5 mg/mL) was added to coat the surface for 6 h in an incubator under a humidified atmosphere of 5% CO_2_ in air at 37 °C. Afterwards, the microgels dispersion was removed and the microgels-coated culture dish was washed three times using phosphate-buffered saline (PBS).

### 3.4. Confocal and Scanning Electron Microscopy Imaging of Microgel-Coated Surfaces 

A coverslip was first coated by using the above described method. For confocal microscopy imaging (NikonC1si CLSM, equipped with a standard fluorescence detector, Nikon, Japan), a stock solution of fluorescein sodium salt (Sigma-Aldrich) in deionized water (0.9 mg/mL) was prepared. Into 1 mL (5 mg/mL) microgels coating dispersion, 30 μL of the stock solution was added. The mixture was incubated for 30 min at room temperature. The excitation and emission wavelengths were 460 nm and 515 nm, respectively. For scanning electron microscopy imaging (SEM, FEI Quanta 400 FEG, 15 kV), a microgels dispersion (5 mg/mL) was loaded onto a silica wafer and dried at room temperature overnight before its imaging.

### 3.5. Isolation and Culture of MSCs

The bone marrow–derived MSCs were isolated from four-week-old C57BL6 mice using the established protocol. Briefly, the femora of mice were taken and the connective tissue was removed. Both ends of the femora were cut and the bone marrow was flushed out with PBS from the femoral cavity. The cells were purified by controlling the digestion time. MSCs were maintained in α-MEM supplemented with 10% fetal bovine serum (Gibco), 1% penicillin/streptomycin and 1% glutamine (Gibco). MSCs were incubated under a humidified atmosphere of 5% CO_2_ in air at 37 °C and passaged every five to six days using 0.25% (*w*/*v*) trypsin-EDTA solution (Gibco).

### 3.6. In Vitro Osteogenic Differentiation of MSCs

For the osteogenic differentiation, ~3 × 10^4^ MSCs were placed in each well of a 24-well plate. On the next day, the osteogenic medium (α-MEM composed of 10% fetal bovine serum, 1% penicillin/streptomycin, 1% glutamine, 50 μg/mL l-ascorbic acid (Sigma), 10 mM β-Glycerophosphate (Sigma) and 10^−7^ M dexamethasone (Sigma-Aldrich)) was supplemented and changed every 3 days. The cells were harvested at Day 14 or 21, respectively.

### 3.7. Alkaline Phosphatase and Alizarin Red S Staining

The culture medium was first removed before the cytochemical staining. The cell layer was rinsed with PBS and fixed by 10% formalin for 15 min at room temperature. After removing the fixing solution, the cell layer was washed by deionized water before the one-step NBT/BCIP solution (Thermo Scientific, Waltham, MA, USA) or the Alizarin Red S staining solution (2% aqueous solution, pH = 4.1~4.3) was added. The staining process was monitored and run for up to 1 h. Afterwards the staining solution was aspirated and the cell layer was washed by deionized water for three times followed by air dry. 

### 3.8. Immunofluorescence Microscopy Observation

MSCs were fixed, permeabilized with 0.1% Triton X-100 (Sigma), and blocked with 5 wt % BSA (Sigma) for 1 h. The samples were incubated first with primary antibodies against F-actin overnight at 4 °C and then with secondary antibodies (1:1000; Invitrogen) for 1 h before they were washed by the DAPI solution (1 μg/mL; Sigma) for 10 min at room temperature. The samples were imaged using a fluorescence microscopy (Nikon Ti-E, Minato, Tokyo, Japan).

### 3.9. Real Time Reverse Transcription-Polymerase Chain Reaction (RT-PCR)

RNA was extracted using the RNAiso Plus (TaKaRa, Katsushika, Tokyo, Japan). 1 μg RNA was reversibly transcribed to cDNA using the 10 μL PrimeScript RT Master Mix (TaKaRa) by the recommended protocol. The product (cDNA) was diluted to 30 μL. 1 μL of the cDNA solution was further used as a template for real time PCR in an ABI7900HTReal Time PCR system. The expression of mRNA was determined using the standard SYBR Premix Ex Taq kit (TaKaRa).

### 3.10. Statistical Analysis

The results of triplicate experiments were reported as mean ± standard deviation. Student’s *t*-test and analysis of variance (ANOVA) were used to determine the statistical significance, and *p* < 0.05 was considered significant. 

## 4. Conclusions

Our current study reveals that a layer of thermally sensitive poly(*N*-isopropylacrylamide-acrylic acid) (P(NIPAM-AA)) spherical microgels coated on a cell culture substrate not only makes the cell attachment and digestion of mesenchymal stem cells (MSCs) easier, but also affects the F-actin alignment and the cell shape, i.e., from an original spindle morphology to a more elongated and stretched shape with thicker and denser actin filaments. Even without any inducing factor, the microgel-coated substrate enhances the expression of some osteogenesis-related genes but cannot induce mineralization. In synchronizing with inducing factors, the MSCs on the microgel-coated substrates show a positive signal in Alizarin Red S staining and a higher expression level of osteopontin, indicating that the cytoskeleton alignment altered by the microgel coating eventually affects the differentiation of the MSCs.
